# Influence of Errors in Tactile Sensors on Some High Level Parameters Used for Manipulation with Robotic Hands

**DOI:** 10.3390/s150820409

**Published:** 2015-08-19

**Authors:** José A. Sánchez-Durán, José A. Hidalgo-López, Julián Castellanos-Ramos, Óscar Oballe-Peinado, Fernando Vidal-Verdú

**Affiliations:** 1 Departamento de Electrónica, ETSI Informática Universidad de Málaga, Andalucía Tech, Campus de Teatinos, Málaga 29071, Spain; E-Mails: jsd@uma.es (J.A.S.-D.); jahidalgo@uma.es (J.A.H.-L.); julian@elca.uma.es (J.C.-R.); oballe@uma.es (O.O.-P.); 2 Instituto de Investigación Biomédica de Málaga (IBIMA), Málaga 29010, Spain

**Keywords:** tactile sensor errors, tactile image moments, robotic hands

## Abstract

Tactile sensors suffer from many types of interference and errors like crosstalk, non-linearity, drift or hysteresis, therefore calibration should be carried out to compensate for these deviations. However, this procedure is difficult in sensors mounted on artificial hands for robots or prosthetics for instance, where the sensor usually bends to cover a curved surface. Moreover, the calibration procedure should be repeated often because the correction parameters are easily altered by time and surrounding conditions. Furthermore, this intensive and complex calibration could be less determinant, or at least simpler. This is because manipulation algorithms do not commonly use the whole data set from the tactile image, but only a few parameters such as the moments of the tactile image. These parameters could be changed less by common errors and interferences, or at least their variations could be in the order of those caused by accepted limitations, like reduced spatial resolution. This paper shows results from experiments to support this idea. The experiments are carried out with a high performance commercial sensor as well as with a low-cost error-prone sensor built with a common procedure in robotics.

## 1. Introduction

Progress in new technologies in instrumentation and robotics has resulted in the development of commercial products like artificial robotic hands that are equipped with tactile sensors [1,2]. These tactile sensors are based on different technologies, mainly resistive or capacitive, but they share common errors like hysteresis, mismatching (this term is similar to the term “fixed pattern noise” used for other 2D-matrix sensors such as digital imaging sensors) or drift. Therefore, calibration procedures should be carried out to minimize the influence of these non-idealities. This is commonly done when the sensors are used as instruments to characterize contact interfaces, so they are not part of the examined system. To do such a calibration, a pneumatic device exerts uniform pressure on the surface of the sensor and the correction parameters are obtained [3,4]. However, this procedure has some limitations. Firstly, the sensor is calibrated on a flat surface, but the contact interface may have a different shape, and interferences from bending arise after calibration, once the sensor is placed on the system under test. Secondly, the response can vary depending on the compliance of the contacting objects, and the calibration should take this into account [5]. Thirdly, the calibration should be repeated often, because the sensor response changes significantly with time and surrounding environments; the previous procedure could mean the sensor needs to be dismounted and mounted again on the robotic hands, which could be cumbersome or even not possible.

Nevertheless, although tactile sensors may have poor behavior in terms of errors from the taxel point of view, their limitations are not so serious if we see them as a whole matrix, and from the point of view of their use in applications. These applications often preprocess the tactile image to obtain only a few key parameters such as input for further control or recognition tasks. For instance, this is done to derive the moments of the tactile image, which provide information about the shape and dimensions of the contact and its location related to the tactile pad, and the contact force [6,7]. Specifically, the zero order moment provides the contact force. The first order moments provide the centroid, which lets it be known if the contact is made with the center of the fingertip or grip; a key problem in robot grasping is that of positioning the manipulator contacts [8]. Second order moments provide information about the dimensions and orientation of the object and are used in complex manipulative tasks like opening a door [9]. An ellipsoid can be obtained from these tactile moments whose location, shape, size and orientation resembles the related contact properties. These arguments could be extended to other tasks like recognition of objects. Recognition can be achieved swiftly as if it were being visualized, analyzing and processing an image of high enough resolution, but a more practical approach consists in exploring the objects making successive contacts with the robotic hand [10].

The described preprocessing could even be mandatory to adapt to the limited throughput of the communication buses and real time processing capability. These tasks require intensive transmission and processing of data. Communication is usually done through buses like USB and Ethernet, while other buses that guarantee a certain latency like CAN are dedicated to real time critical tasks, for instance manipulative ones [11,12]. To assure this latency, CAN bus limits the length of the data field of its messages to 8 bytes. Taking into account that a hand with three fingers equipped with the DSA 9200 tactile sensor system from Weiss Robotics (sensors 9205 and 9210) [2,13] has 462 taxels and that there are usually more devices on the bus, it can take several milliseconds to send the whole tactile image [14]. Moreover, this raw data must be processed by the central unit that performs the control and modifies the grasp forces accordingly. The information regarding the shape of the contact area, the friction and direction of fingertip forces begins to shape fingertip force output within about 70 ms in human manipulation as stated in [15]. Therefore, taking into account the other data sources and control tasks that should be attended, pre-processing of raw data must be done to reduce the information to communicate and the processing load of the central unit.

**Figure 1:**
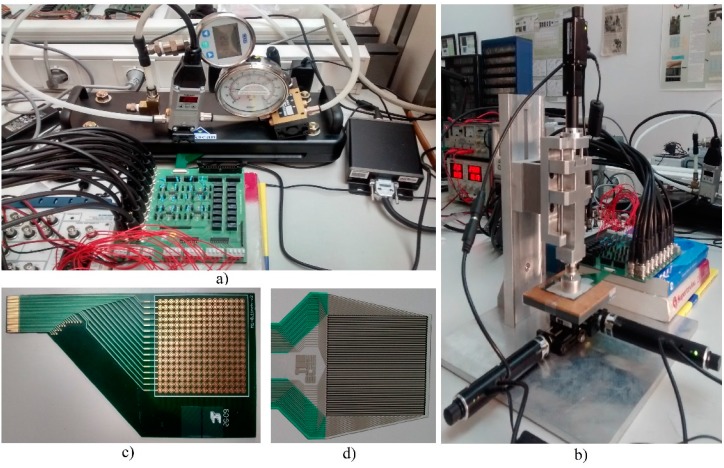
(**a**) Pneumatic commercial setup Tekscan PB100E; (**b**) Motorized stage; (**c**) PCB based tactile sensor; (**d**) Commercial Sensor Tekscan 5051/P1/3056T1/20.

This paper shows results from experiments focused on the evaluation of the influence of tactile sensor limitations on the above mentioned reduced set of parameters. Specifically, on the tactile image moments or the equivalent variables that define the associated ellipsoid. An error prone piezoresistive tactile sensor is used to see the impact of hysteresis and drift (see Figure 1c). This sensor is made by placing a sheet of sensitive material on an array of electrodes, a quite common procedure in sensors for robotic hands [10,16]. Another commercial sensor is also used, mainly to see the effect of mismatching and limited resolution (see Figure 1d [17]). This commercial sensor has good linearity and low hysteresis and also a high resolution. This good performance allows the comparison of the influence of different parameters isolated to others such as non-linearity or hysteresis. Specifically, the resolution can be changed by software and the variation of the target parameters estimated. Mismatching or differences in the response of different taxels can also be seen to be quite isolated from other interferences using commercial equipment to calibrate the sensor and equalize the output of all taxels. Although the subject is complex and this paper presents results from a limited number of experiments, it is enough to provide clear indications about the weight of these non-idealities in the sensor performance for the above mentioned applications.

The rest of the paper is structured as follows: in Section 2, the experimental set up and materials used to carry out the experiments are described; Section 3 provides performance data from the two tactile sensors used in the tests; Section 4 presents the methodology of the subsequent experiments and Section 5 shows the obtained results and associated discussion. Finally, Section 6 summarizes the main conclusions derived from the results.

## 2. Experimental Setup

Two setups have been used to carry out the experiments and characterize the sensors. One of them obtains readings of the whole tactile matrix under the same uniform pressure. It is based on a pneumatic commercial equilibration/calibration device (Tekscan PB100E [17]; see Figure 1a). The sensor is laced in a slot where one side is rigid and the other is a flexible wall of a chamber. When the chamber is pressurized the wall exerts an even pressure on the sensor. Since the device is intended to be used to study the hysteresis, it has been customized to perform loading-unloading cycles as defined from the computer interface. It has been achieved by adding an electro-valve that allows the flow from a compressor until the pressure reaches the desired value. The other way is also possible by opening the electro-valve to reduce the pressure in the chamber. The electro-valve is the Pneumax 171E2N.T.D.0009 [18] and it is controlled by an application on a computer.

**Figure 2:**
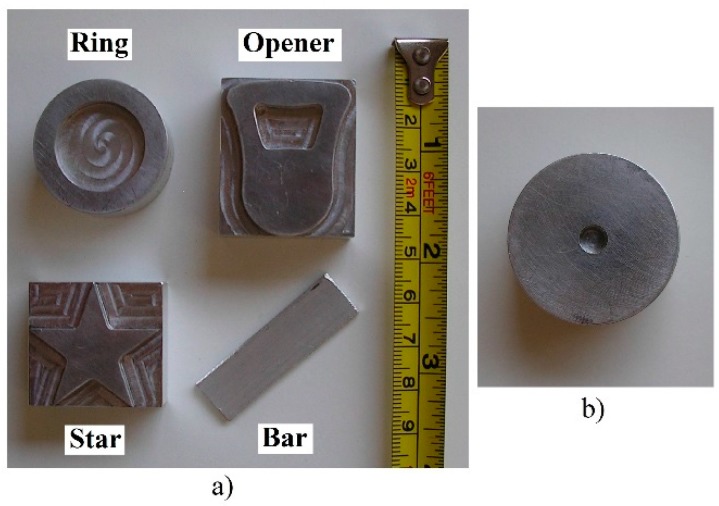
(**a**) Objects used to carry out the experiments (from left to right and top to bottom: ring, opener, star, and bar); (**b**) Hole atop the ring object to insert the tip of the force sensor probe.

Moreover, a few metal pieces with different shapes were used to achieve different pressure patterns on the tactile sensors. Figure 2a shows them and their dimensions. A motorized stage was built to exert a force on the top of these objects once they are placed on the tactile sensor (see Figure 1b). The stage has a T-NA08A50 linear actuator to move along the z axis and two T-LA60A actuators for displacements along x and y axes (all of them from Zaber Technologies [19]). A piston with a spring was also added in the z axis to enlarge the dynamic range. The sensor Nano17 from ATI Industrial Automation [20] was also added at the tip of the motor in the vertical direction, to register the actual force that is exerted on the objects and then on the sensor. A hole was made at the center of mass of the objects (see Figure 2b) and the tip of the force sensor probe is inserted into it. In this way a pressure equal to the force registered by the sensor divided by the contact area between the object and the sensor is applied to the sensor.

Regarding the electronics used to acquire the tactile sensor output, the I-Scan commercial handle [21] is employed with the sensor from Tekscan. With respect to the other custom sensor, the readings of the whole tactile array are registered by means of well-known interface electronics [22]. Since the goal is not the dynamic evaluation, the electronics was designed to achieve a good static performance, so electro-mechanic relays were used to implement the switches to select the rows as the array is scanned. In this way the errors due to the non-zero on resistance of the switches are minimized. The data acquisition is done with the NI-USB 6259 device from National Instruments. Sixteen analog inputs are multiplexed to scan up to 16 × 16 taxels in our testing platform.

## 3. Tactile Sensors

The results of this paper are obtained from tactile sensors based on a piezo-resistive principle. One of them is built with a common procedure that consists in placing a layer of sensitive material on an array of electrodes, usually made on a flexible printed circuit board (see Figure 1c). The sensitive electroactive material is a conducting polymer in the case of the sensor of this paper [22]. The resistance between two electrodes associated to each taxel changes when the force against the taxel increases.

The second sensor is the 5051/P1/3056T1/20 from Tekscan (see Figure 1d), it is also piezoresistive but fabricated with a screen printing technology. It has a symmetrical structure in the z axis, where two identical layers, with conductive tracks plus sensitive ink on them, are put in contact in a column and row fashion, and each cross of the array is a taxel.

In the following section, some performance data of both sensors are provided. These data are obtained with the set-up shown in Figure 1a that exerts a uniform pressure on the sensor surface so it allows the output from all taxels to be read, in the sensor under similar conditions. The results in this section are not actually a complete characterization of the sensors since that is not the goal of this paper. The only purpose it serves here is to gain a better knowledge of them, which will help to understand the readings obtained later in the experiments.

### 3.1. Hysteresis

**Figure 3:**
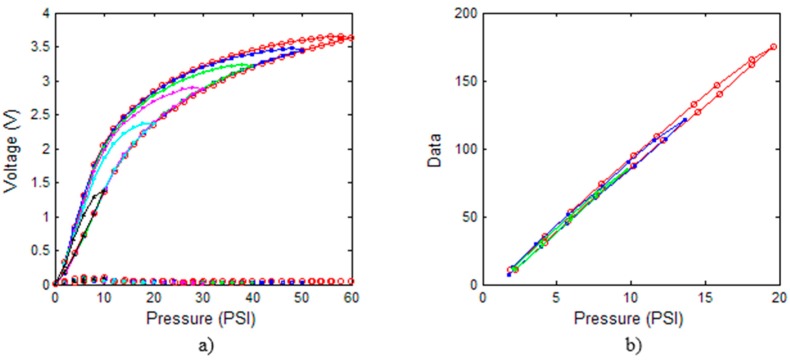
(**a**) Average output and standard deviation (at the bottom) from the PCB based sensor; (**b**) Average output from the commercial sensor.

Tactile sensors suffer from hysteresis, *i.e.*, their response under a certain pressure actually depends on previous pressure values. To quantify the hysteresis, the pressure on the sensor is increased and then decreased, and the maximum deviation between the obtained responses in both directions is taken as the hysteresis error. Figure 3a,b show averaging of the output of all taxels of the sensors in Figure 1c,d respectively, when the pneumatic equilibration/calibration device in Figure 1a is controlled from the computer to carry out six consecutive loading-unloading cycles. For the PCB sensor they are as follows: 0 psi → 60 psi → 0 psi → 50 psi → 0 psi → 40 psi → 0 psi → 30 psi → 0 psi → 20 psi → 0 psi → 10 psi → 0 psi in increments of 2 psi. The time interval between the new pressure level being exerted and the voltage output being registered by the acquisition board is 5 s. The previous procedure is done 10 times and the average is computed and displayed in Figure 3a (the standard deviation is also shown at the bottom of the same figure). A few captions for intermediate values of the outer cycle are also shown in Table 1. Note also the slight difference between the real value of the pressure for frames that are presumably under the same conditions. This is because the pressure changes must always be made in the same sense, increasing or decreasing its value depending on the path in the cycle, and it is also due to the limited control capability of the system. Also note the mismatching or variation of the response of different taxels.

**Table 1:** Tactile images from the PCB based sensor obtained for a uniform pressure that follows and increasing-decreasing cycle.

UP →	4.1 PSI	9.98 PSI	19.92 PSI	29.88 PSI	39.91 PSI	49.98 PSI	59.81 PSI
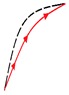	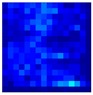	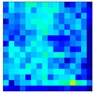	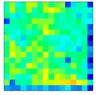	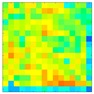	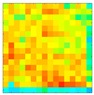	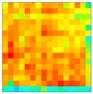	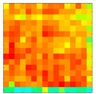
DOWN →	3.91 PSI	9.84 PSI	19.82 PSI	29.85 PSI	39.87 PSI	49.96 PSI	
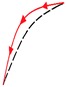	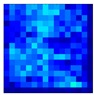	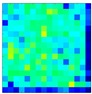	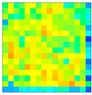	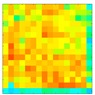	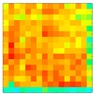	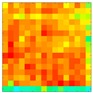	
Difference →	∆	∆	∆	∆	∆	∆	
	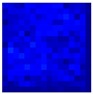	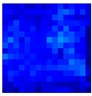	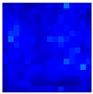	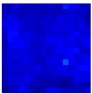	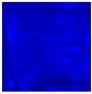	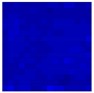	
Mean (V):	0.37	0.61	0.47	0.35	0.26	0.17	
σ (V):	0.20	0.25	0.17	0.13	0.09	0.05	
σ/Mean (V):	0.54	0.41	0.36	0.38	0.36	0.33	

The same previously described procedure is carried out with the sensor 5051/P1/3056T1/20 from Tekscan in Figure 1d, but now the cycles are: 0 psi → 20 psi → 0 psi → 15 psi → 0 psi → 10 psi → 0 psi → 5 psi → 0 psi, because the pressure rating of the sensor is 20 psi. The raw data are not equilibrated nor is calibration taken to obtain the curves in Figure 3b. These data are provided by the I-Scan acquisition system [21] that encodes the pressure in a scale of 256 levels (8 bits).

Note that the low cost sensor based on a flexible printed circuit board presents a hysteresis error much larger than that observed in the commercial one in Figure 1d. Moreover, the linearity is also much higher in the latter than in the first sensor. This behavior is not due only to the PCB technology, but also to other factors like size and geometry of the electrodes; mechanical properties of the sensitive layer atop; and adherence and roughness of the interface between both [22,23]. Nevertheless, since the goal of this paper is to compare the impact of the hysteresis with that from other error sources, it may be appropriate to take this simple sensor despite its limited performance from the taxel point of view. We will discuss if this poor performance is translated at system level in a tolerable amount or not.

### 3.2. Drift

**Figure 4:**
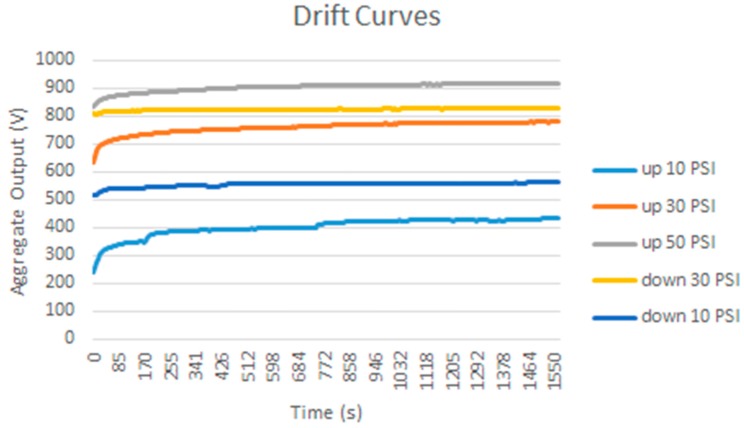
Drift of the *Aggregate Output* registered by the PCB based sensor for different increments and decrements of the uniform force on its surface.

Another common source of errors in tactile sensors is drift, or change, of the sensor output over time when the pressure exerted on it does not change. A test was carried out with the calibration/equilibration set-up in Figure 1a described in Section 2 to estimate the drift of the custom sensor Figure 1c. The pressure is changed in large increments and remains stable (as long as the regulation system achieves stability) for a long period before changing again to the next value. Pressure transitions are increments or decrements, specifically the results in Table 2 correspond to the sequence 10 psi → 30 psi → 50 psi → 30 psi → 10 psi. There are 255 samples taken every 5 s and the reading takes 1 s, therefore the time elapsed to estimate the drift is around 25 min. A rather large drift is observed, and it is not the same for all taxels, as can be seen in the difference as well as in the histograms displayed in Table 2. The drift is larger for small pressure values as Figure 4 depicts, and the mismatching between the drift of different taxels is also larger for these pressures. Nevertheless, the regulation of the pressure is also more difficult in these cases and this causes the slight knee points of the curves. Drift is also lower for pressure decrements, and dispersion between taxels is also smaller. Similar experiments were carried out with the commercial sensor in Figure 1d and the observed drift was less than that observed for the custom PCB based sensor, so again the latter is a good choice to observe the impact of drift in the above mentioned high level application parameters.

**Table 2:** Tactile images from the PCB based sensor obtained for different increments and decrements of the uniform force on its surface.

Pressure:	10 PSI	30 PSI	50 PSI	30 PSI	10 PSI
First Frame →	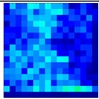	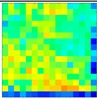	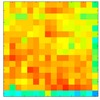	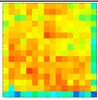	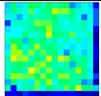
Mean (V):	0.94	2.47	3.25	3.17	2.02
σ (V):	0.53	0.61	0.45	0.49	0.68
Last Frame →	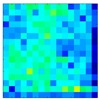	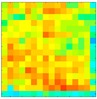	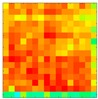	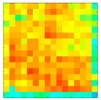	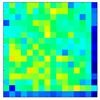
Mean (V):	1.69	3.04	3.58	3.23	2.19
σ (V):	0.53	0.46	0.44	0.47	0.64
Absolute Distance →	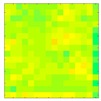	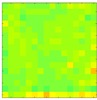	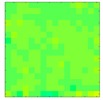	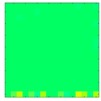	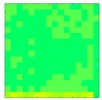
Mean (V):	0.76	0.57	0.33	0.06	0.17
σ (V):	0.24	0.22	0.10	0.09	0.14
Drift Curve →	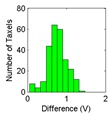	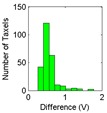	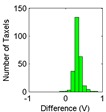	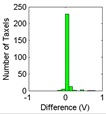	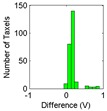

### 3.3. Mismatching

Since they are matrix sensors, a noticeable variation, or mismatching, between the response of different taxels of the same tactile sensor is observed. Therefore, calibration should be made to equalize the sensor output. This can be achieved with the pneumatic device in Figure 1a. It is worth highlighting however that this calibration has quite limited duration and can be altered if the shape of the sensor changes. A simple experiment can demonstrate this fact. Table 3(a) displays the output of the Tekscan 5051/P1/3056T1/20 sensor when a pressure of 15 psi is exerted on its surface, while Table 3(b) shows the same output once the sensor is equilibrated using a procedure recommended by the vendor. The sensor is then removed from the slot and it is waved in the air so it bends softly, then it is inserted again into the device. Now the response in Table 3(c) is not as uniform as that in Table 3(b) after the equilibration. If the sensor is placed on a rigid surface and different objects are put on it (like a star in the case of Table 3(d)), the response is again somewhat altered as observed in Table 3(e) when it is inserted in the calibration device. Taking into account that tactile sensors in robotics are usually mounted on curved surfaces, and therefore not commonly calibrated after fabrication, and are covered with flexible protective layers that suffer from wear, it can be assumed that a quite large mismatching remains in the sensor output and it is accepted in practical applications.

**Table 3:** (**a**) Raw output; (**b**) Equilibrated output; (**c**) Output from the equilibrated sensor after it is waved in the air; (**d**) Output when a force is exerted against the sensor with the star object; (**e**) Output after previous manipulation.

(a)		(b)		(c)		(d)		(e)	
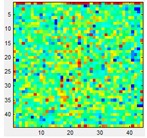		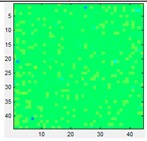		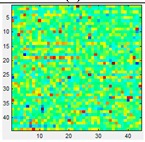		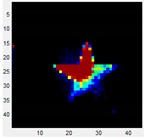		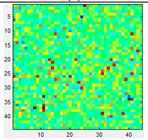	
Pressure:	15 PSI	Pressure:	15 PSI	Pressure:	15 PSI	Force:	4 Kg	Pressure:	15 PSI
Mean:	126.66	Mean:	129.57	Mean:	128.09			Mean:	127.28
Std.:	27.87	Std.:	2.89	Std.:	23.62			Std.:	21.60
Std.*100/FS	10.93%	Std.*100/FS	1.13%	Std.*100/FS	9.26%			Std.*100/FS	8.47%
Std/Med	0.22	Std/Med	0.022	Std/Med	0.18			Std/Med	0.17

### 3.4. Spatial Resolution

The PCB based sensor in Figure 1c has 16 × 16 taxels and the distance between centers in the row and column directions, or spatial resolution, is 2.54 mm. The commercial sensor in Figure 1d has 44 × 44 taxels and a spatial resolution of 1.27 mm. The high resolution of the latter will allow to assess the influence of limited resolution to be assessed, since we can merge taxels to change the resolution and still have one comparable to those reported by many tactile sensors for robotic hands, for instance 3.4 mm for the DSA 9205 sensor from Weiss Robotics [13].

## 4. Methodology

### 4.1. Parameters for Performance Assessment

**Figure 5:**
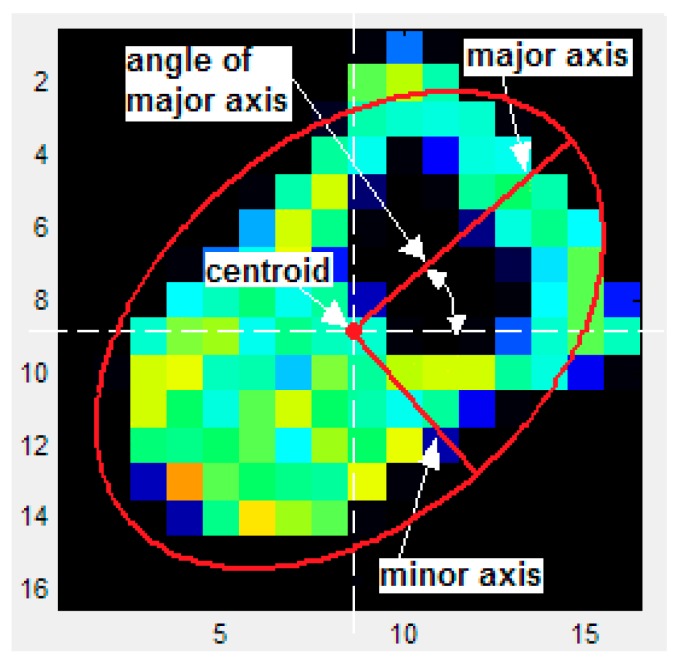
Parameters of the ellipse used to describe the object in manipulation tasks.

Figure 5 shows a set of parameters that provide useful information for manipulative, recognition or control tasks in general, as mentioned above.

They are related to the ellipsoid location, shape and orientation and are obtained from the moments of the tactile image computed as:

(1) $$M_{ij}=\sum_{x=1}^{N}\sum_{y=1}^{M}x^{i}y^{j}f(x,y)\;i,j>0$$

where f(x,y) is the output of the taxel with coordinates (x,y), and *N* and *M* are the number of rows and columns of the sensor array, respectively. The coordinates of the centroid (*X*, *Y*) in Figure 5 are obtained from these moments (see Table 4) and then it is possible to express the moments referred to this centroid, and therefore independently of the ellipsoid location, in the so called central moments as:

(2) $$CM_{ij}=\sum_{x=1}^{N}\sum_{y=1}^{M}{\left(x-X\right)}^{i}{\left(y-Y\right)}^{j}f(x,y)\;i,j>0$$

**Table 4:** Parameters obtained from the processing of the tactile image and used commonly in control tasks.

Parameter	Equation	Variation from *a* to *b* (%)
*Centroid*	$X=\frac{M_{10}}{M_{00}}$	$\frac{\sqrt{{\left(X_{a}-X_{b}\right)}^{2}+{\left(Y_{a}-Y_{b}\right)}^{2}}}{L}\times 100$
	$Y=\frac{M_{01}}{M_{00}}$	
*Major Axis*	$2\cdot \sqrt{\frac{I_{1}}{CM_{00}}}$	$\frac{\left|value(a)-value(b)\right|}{L}\times 100$
*Minor Axis*	$2\cdot \sqrt{\frac{I_{2}}{CM_{00}}}$	
*Angle*	$\tan^{-1}\left(\frac{\max(I_{1},I_{2})-CM_{20}}{CM_{11}}\right)$	$\frac{\left|value(a)-value(b)\right|}{180º}\times 100$
*Area*	$\sum_{x=1}^{N}\sum_{y=1}^{M}b(x,y)$	$\frac{\left|value(a)-value(b)\right|}{L^{2}}\times 100$
*Aggregate Output*	$\sum_{x=1}^{N}\sum_{y=1}^{M}f(x,y)$	$\left|\frac{value(a)-value(b)}{\left(\frac{value(a)+value(b)}{2}\right)}\right|\times 100$

Table 4 shows the expressions used to compute the parameters labeled in Figure 5 besides of the *Area* and *Aggregate Output* from Equations (1) and (2). Moreover, I_1_ and I_2_ in Table 4 are the eigenvectors of the covariance matrix of the image [24] obtained as:

(3) $$I_{k}=\frac{\left(CM_{20}+CM_{02}\right)-{\left(-1\right)}^{k}\sqrt{{\left(CM_{20}-CM_{02}\right)}^{2}+4CM_{11}^{2}}}{2}\;k=1,2$$

and *b(x,y)* is a thresholding function to obtain a binary image from the original one. Its value is b(x,y)=1 if f(x,y)>0 and 0 otherwise.

The column on the right of Table 4 shows the variation of the parameters as computed to obtain the results of this paper. Most of them are expressed as relative changes with respect to the full scale. However, the full scale value is not obvious and has to allow comparison between results obtained with different sensors and objects as well as to suit its meaning as maximum reference value. Since the objects in Figure 2a were built to fit in the sensor of Figure 1c while taking advantage of its spatial resolution, *i.e.*, making them large considering the size of a taxel, the length of the square size L = 40.64 mm of this sensor was chosen as full scale reference for the parameters related to size or translation, while 180° was chosen as full scale value for the parameter *Angle*.

Finally, note that the variation of the parameter *Aggregate Output* is computed as relative to the expression (*value (a)* + *value (b)*)/2 instead of a full scale figure. The reason is that the outputs of the sensors in Figure 1c,d have different units, as can be seen in Figure 3. Moreover, the experiments do not always have the same force range because the output of some taxels is saturated depending on contact area. Therefore, the best way to provide meaningful results valid to make comparisons is that displayed in Table 4. Nevertheless, note that this figure is large for small values of (*value (a)* + *value (b)*)/2, and this should to be taken into account in the analysis of the results of the experiments.

### 4.2. Procedure

The setups in Figure 1a,b and the objects in Figure 2a are used, as mentioned in Section 2, to exert different pressure patterns on the tactile sensors. A piece of fabric has been added between the objects and the sensors to improve the contact. The goal is to evaluate the change of the parameters in Table 4 due to hysteresis, drift, mismatching and limited resolution. To assess the influence of hysteresis, the set up in Figure 1b has been used to exert an increasing and then decreasing force on the objects in Figure 2, once placed atop of the sensor. The ellipsoid parameters in Section 4.1 are then computed for every tactile image captured along the cycle and the results from frames corresponding to similar forces on the objects are compared. The variation of these parameters for the same load on the sensor is found in a loading-unloading cycle.

To estimate the impact of drift, the change of the parameters in Table 4 is registered when the load is kept in time. The set up in Figure 1b was used to exert a force atop of each object and the sequence 10 N → 20 N → 30 N → 40 N → 30 N → 20 N → 10 N (in the case of the bar the maximum possible force was 38 N) is followed. 255 samples were taken in 25 min. Similar experiments were carried out with the 5051/P1/3056T1/20 from Tekscan, where 900 samples were taken in 30 min. Although the drift of the latter is smaller than that of the former, it is not negligible and it is worth considering its effect on this study.

Regarding mismatching, since it is not possible to see its effects experimentally, we can use a mixed or indirect method to evaluate it. This consists in taking the output for ten uniform pressures, 2.1, 4.3, 6.2, 8.3, 9.7, 12.3, 13.7, 16.2, 17.7 and 20.1 psi, with the pneumatic set-up in Figure 1a. For each pressure, the output is equilibrated and the result is also saved. In this way we have ten raw plus ten equilibrated tactile images of a uniform pressure against the sensor surface. To obtain the data corresponding to different objects or geometries, a binary mask is applied to the previous tactile images. This mask is obtained from readings of the different objects on the sensor.

Finally, the last experiments were carried out to see the effect of the limited resolution of the sensor. Again, we try to see the consequence of this limitation on the parameters of Table 4. To do so, we use the commercial sensor to register the sensor output under different forces exerted with the setup in Figure 1b.

**Table 5:** Tactile images and ellipse for the hysteretic cycle with the objects “ring”, “star”, “opener” and “bar” on the PCB based sensor.

UP →	1.98 N	5.95 N	9.90 N	19.82 N	29.79 N	37.79 N
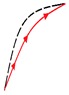	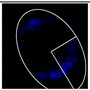	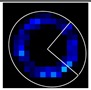	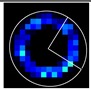	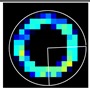	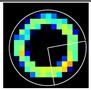	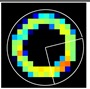
DOWN →	2.06 N	6.02 N	10.06 N	20.10 N	30.12 N	36.48 N
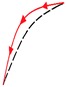	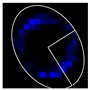	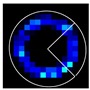	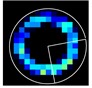	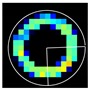	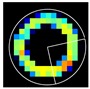	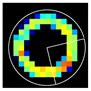
UP →	2.01 N	5.95 N	9.87 N	19.78 N	29.76 N	37.78 N
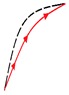	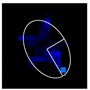	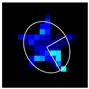	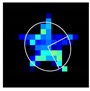	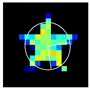	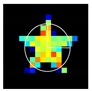	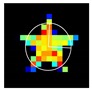
DOWN →	2.09 N	6.06 N	10.06 N	20.09 N	30.13 N	36.64 N
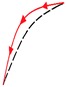	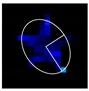	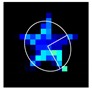	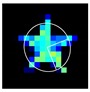	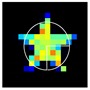	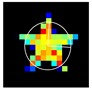	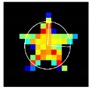
UP →	2.01 N	5.94 N	9.89 N	19.84 N	29.79 N	37.82 N
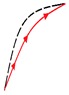	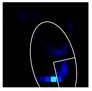	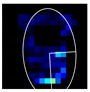	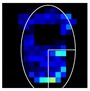	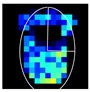	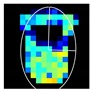	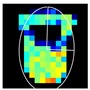
DOWN →	2.13 N	6.06 N	10.05 N	20.10 N	30.18 N	36.48 N
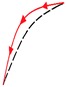	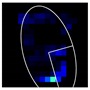	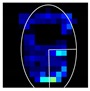	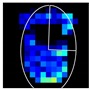	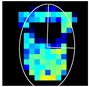	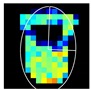	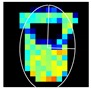
UP →	2.02 N	5.98 N	9.86 N	19.81 N	29.81 N	37.79 N
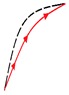	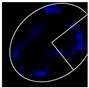	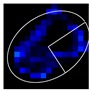	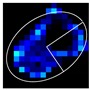	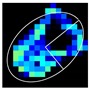	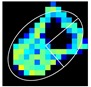	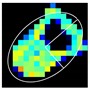
DOWN →	1.98 N	6.04 N	10.08 N	20.11 N	30.16 N	36.72 N
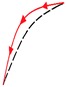	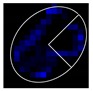	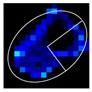	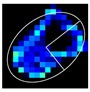	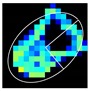	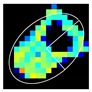	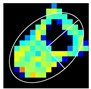
UP →	2.05 N	5.99 N	9.94 N	15.95 N	21.87 N	27.89 N
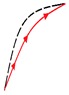	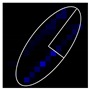	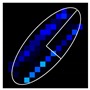	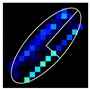	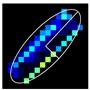	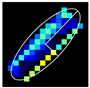	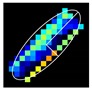
DOWN →	1.97 N	5.92 N	9.98 N	16.01 N	22.03 N	28.00 N
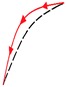	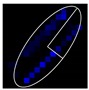	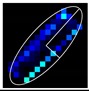	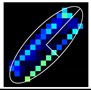	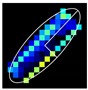	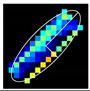	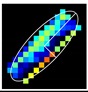

Then the resolution is lowered before computing the parameters in Table 4 by applying a filter to the image so that four adjacent taxels are merged and substituted by a single one whose output is the average of the outputs from the original taxels. This is done for a decrease of 50% and 25% of the spatial resolution.

## 5. Results and Discussion

### 5.1. Hysteresis

**Figure 6:**
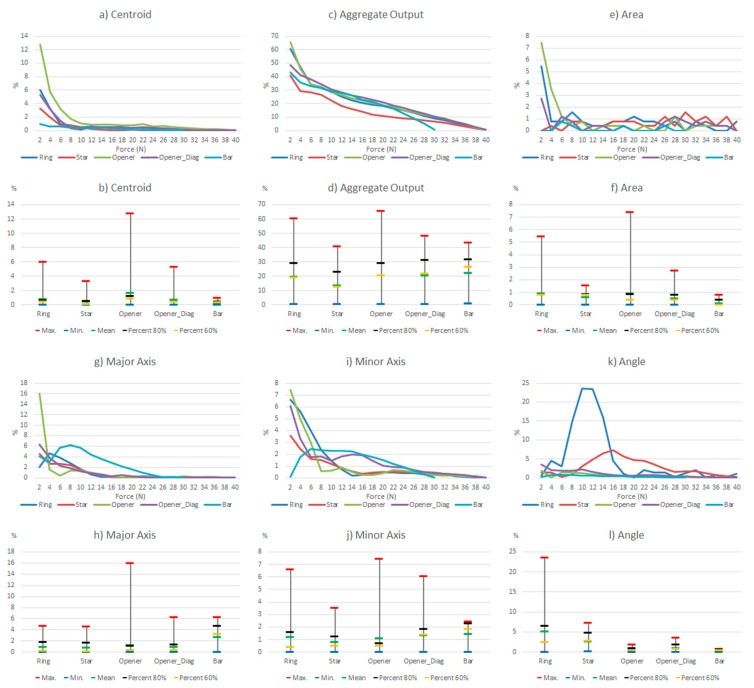
Variations of the system level parameters caused by the PCB based sensor hysteresis.

Since the hysteresis and non-linearity errors of the commercial sensor are very low, this section will show the results of the experiments carried out with the other sensor. Table 5 shows a few selected tactile images out of the whole set registered for the different objects in the above described experiments, as well as their corresponding ellipse. In addition, Figure 6 shows the results of such tests taking the whole set of collected data. The variations of the parameters in Table 4 are displayed in two types of chart for every parameter in the table. The graph at the top shows the variation (as expressed in Table 4) of the parameters along the hysteretic cycle for different objects in Figure 2a. The chart at the bottom summarizes the information in five significant values: maximum, minimum, mean, percentile 80 and percentile 60.

Some features can be observed in Figure 6. Firstly, the variations are larger for small force values. This is mainly because the shape of the object footprint is not yet well defined, as can be seen in Table 5. The largest changes in the centroid location are for low forces, but they are small. The worst behavior is that of the opener in vertical position, worse than the opener in diagonal orientation, so we see that the location of the object in the sensor has influence. This is mainly due to the mismatching between different taxels. Regarding the *Aggregate Output*, note that it is computed in a different way and it is related to the reading and not to a kind of full scale reference. Therefore Figure 6c indicates a quite uniform behavior for changing forces. The area is little affected except for low forces. The variations of the object shape, *i.e.*, the size of the axis, are slightly larger for the bar or the opener-like object, which have an oblong shape. The objects with radial symmetry like the ring and the star show the largest errors in orientation, as indicated by the parameter *Angle*.

### 5.2. Drift

The results for the drift are shown in Table 6 and Table 7 for the object labeled as “opener” and for the PCB based sensor and the Tekscan sensor respectively. It is observed again that the drift is larger in the PCB based sensor and for pressure increments larger than for decrements. It can also be observed that the histograms are more symmetric in Table 6 when compared with those in Table 7.

**Table 6:** Tactile images of the opener taken with the PCB based sensor to see the effect of drift on the parameters of Table 4.

Force (N):	10	20	30	40	30	20	10
First Frame	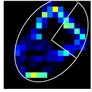	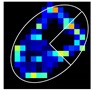	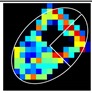	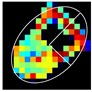	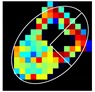	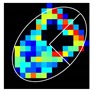	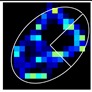
Mean (V):	0.34	0.45	0.86	1.10	1.08	0.81	0.39
σ (V):	0.60	0.73	1.22	1.51	1.49	1.17	0.65
Last Frame	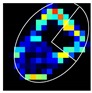	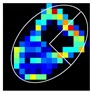	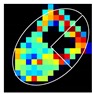	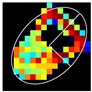	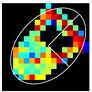	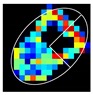	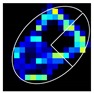
Mean (V):	0.51	0.64	0.99	1.21	1.10	0.84	0.48
σ (V):	0.85	0.96	0.14	1.64	1.51	1.20	0.74
Absolute Distance	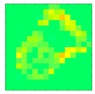	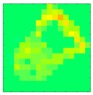	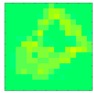	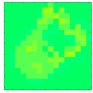	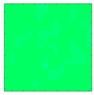	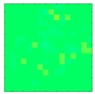	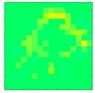
Mean (V):	0.17	0.19	0.13	0.10	0.02	0.02	0.08
σ (V):	0.30	0.31	0.20	0.15	0.04	0.07	0.17
Histogram	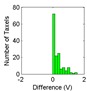	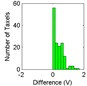	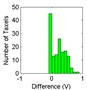	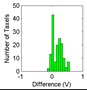	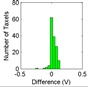	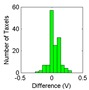	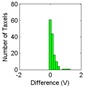

**Table 7:** Tactile images of the opener taken with the commercial sensor to see the effect of drift on the parameters of Table 4.

Force (N):	10	20	30	40	30	20	10
First Frame	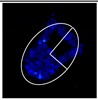	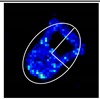	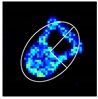	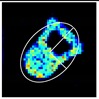	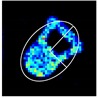	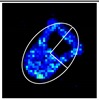	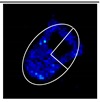
Mean:	4.48	9.41	14.21	19.18	16.00	11.05	5.76
σ:	10.44	20.52	30.31	40.55	33.99	23.86	13.01
Last Frame	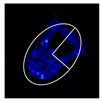	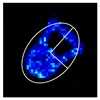	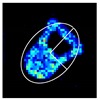	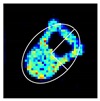	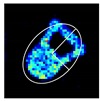	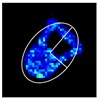	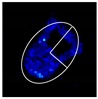
Mean:	4.85	9.89	14.58	19.54	16.18	11.22	5.85
σ:	11.17	21.43	31.00	41.25	34.26	24.23	13.17
Absolute Distance	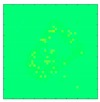	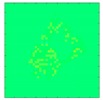	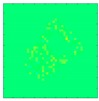	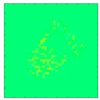	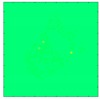	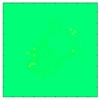	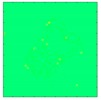
Mean:	0.38	0.47	0.36	0.36	0.13	0.17	0.10
σ:	1.36	1.75	1.85	2.11	0.69	0.90	0.98
Histogram	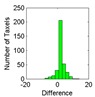	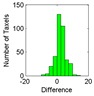	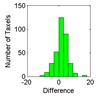	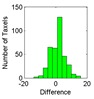	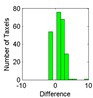	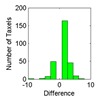	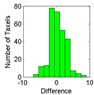

Actually, positive changes at output are registered in Table 6 for almost all taxels, while negative differences are also detected in many taxels of Table 7. This is also noticeable when the absolute distance of both cases in Table 6 and Table 7 are compared.

**Figure 7:**
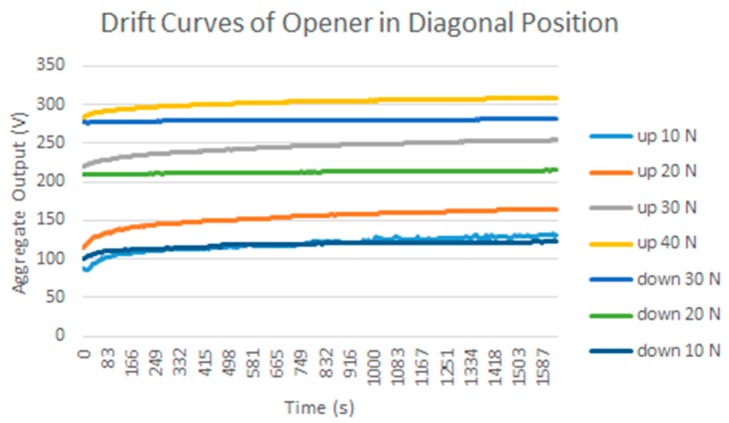
Drift of the *Aggregate Output* registered by the PCB based sensor and the “opener” object atop for different force increments and decrements.

**Figure 8:**
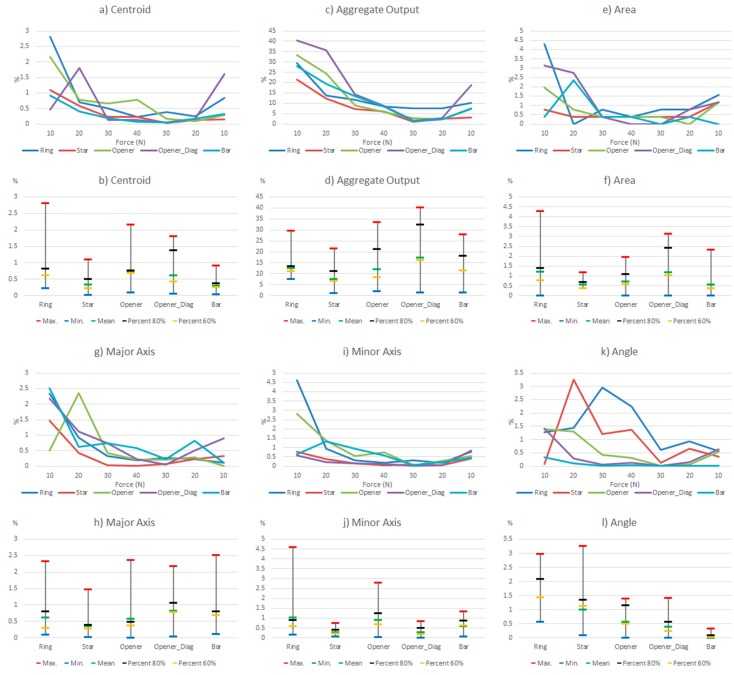
Variations of the system level parameters caused by the PCB based sensor drift.

**Figure 9:**
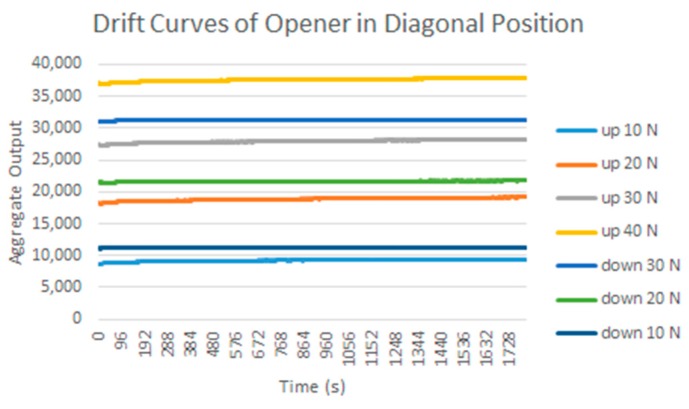
Drift of the *Aggregate Output* registered by the commercial sensor and the “opener” object atop for different force increments and decrements.

**Figure 10:**
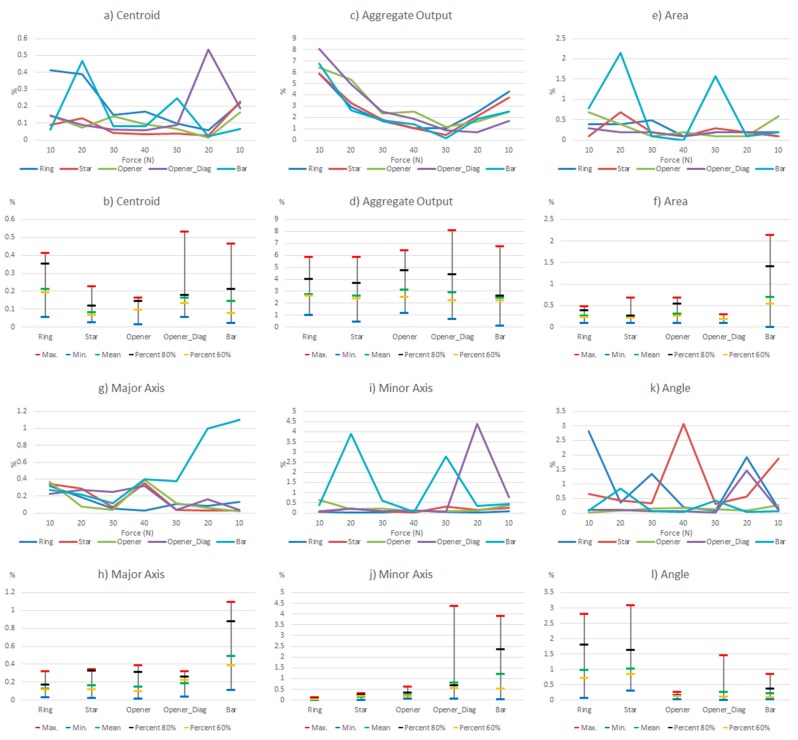
Variations of the system level parameters caused by the commercial sensor drift.

Note that this can mask a larger effect of drift when an average is computed, as done in Figure 7 with respect to Figure 9, or in Figure 8c with respect to Figure 10c, but it can also be seen as a positive compensation effect when the parameters of Table 4 are obtained. This can explain the different behavior observed in Figure 8 and Figure 10.

Generally speaking, a quite clear law is followed in Figure 8 in the sense that the differences decrease for higher pressures and also for decrements, while the curves are more uniform along x axis in Figure 10. An exception is found in the objects in diagonal position whose results are worse, *i.e.*, the orientation of the object in the sensor also affects the result. In both cases a larger difference in the parameter *Angle* is observed for the ring and the star, objects with radial symmetry.

### 5.3. Mismatching

**Figure 11:**
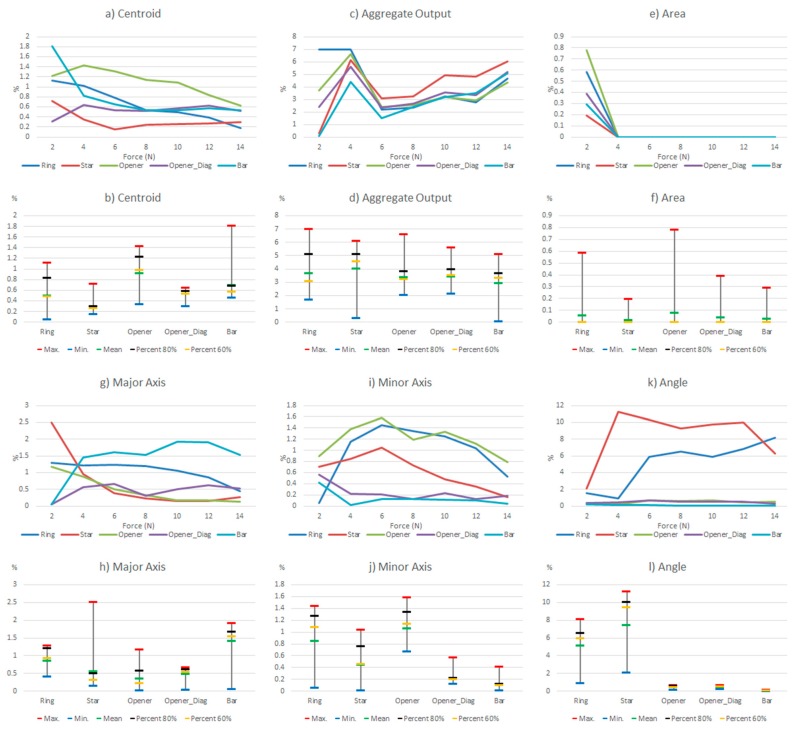
Variations of the system level parameters caused by the commercial sensor mismatching.

Regarding mismatching, the result for the opener is shown in Table 8. The same is done with the other objects and the difference of the parameters in Table 4 is computed as in the previous sections and is displayed in Figure 11. Besides the error magnitude, which will be used later to compare this source of error with the others, no other clear conclusions are extracted from Figure 9. Finally, since the purpose of this paper is to explore the impact of different error sources and not to compare the sensors themselves, the commercial sensor was used for the results of this section because of its very good performance in terms of linearity, hysteresis or drift allowing a certain isolation of the influence of mismatching.

**Table 8:** Tactile images of the opener with and without equilibration.

Pressure:	2.1 PSI	4.3 PSI	6.2 PSI	8.3 PSI	9.7 PSI
Frame Not Equilibrated →	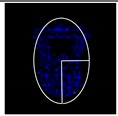	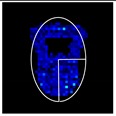	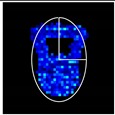	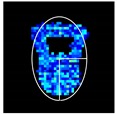	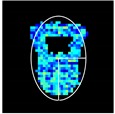
Frame Equilibrated →	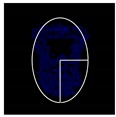	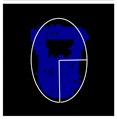	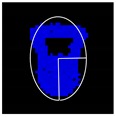	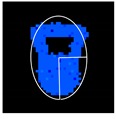	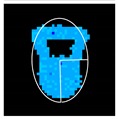
Pressure:	12.3 PSI	13.7 PSI	16.2 PSI	17.7 PSI	20.1 PSI
Frame Not Equilibrated →	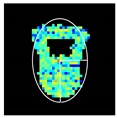	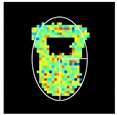	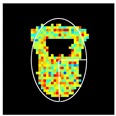	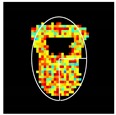	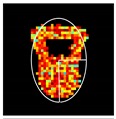
Frame Equilibrated →	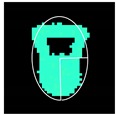	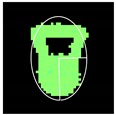	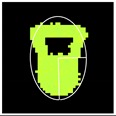	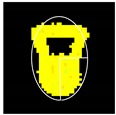	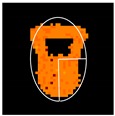

### 5.4. Limited Resolution

**Figure 12:**
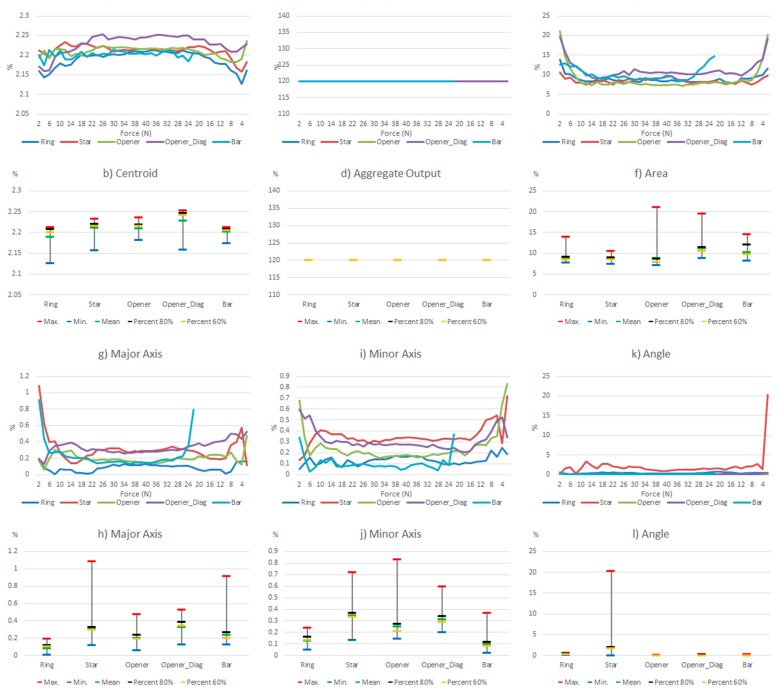
Variations of the system level parameters caused by a 50% decrease of the resolution in tactile images obtained with the commercial sensor.

**Figure 13:**
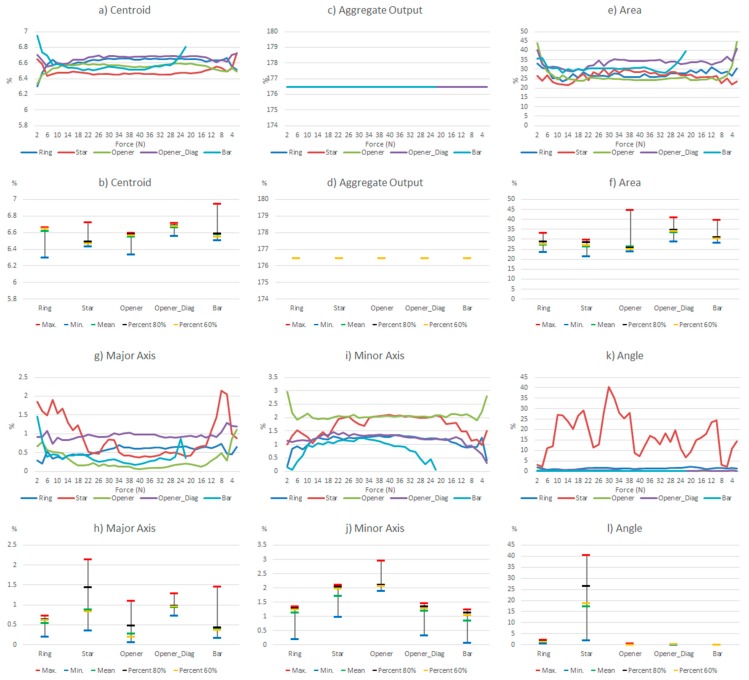
Variations of the system level parameters caused by a 25% decrease of the resolution in tactile images obtained with the commercial sensor.

The results of the experiment described in the final paragraph of Section 4.2 are illustrated in Table 9 for the star object, and the variations of the parameters in Table 4 are calculated and displayed in Figure 12 and Figure 13 for all the objects and two resolution decrements. A quite uniform behavior of the error for different forces is observed in these figures, though it is higher for very low forces (the profile of the object is not well defined yet). Quite large errors are observed for the centroid location, *Aggregate Output* and *Area*, while the error is smaller in the shape estimation (ellipse axes) and very small in the orientation (*Angle*), except in the case of the star. For the latter, a significant impact of the spatial resolution decrement is observed.

**Table 9:** Tactile images of the star obtained with the commercial sensor and resulting images from a decrease in resolution of 50% and 25%.

Force (N)	2.04	6.01	9.92	19.80	29.80	35.75
44 × 44 →	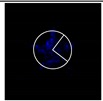	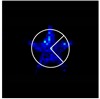	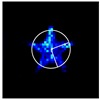	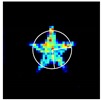	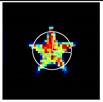	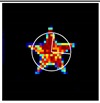
22 × 22 → (50%)	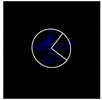	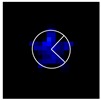	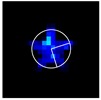	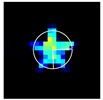	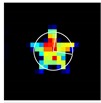	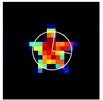
11 × 11 → (25%)	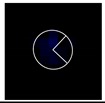	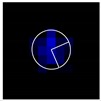	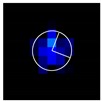	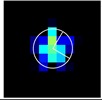	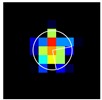	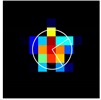

### 5.5. Summary

**Figure 14:**
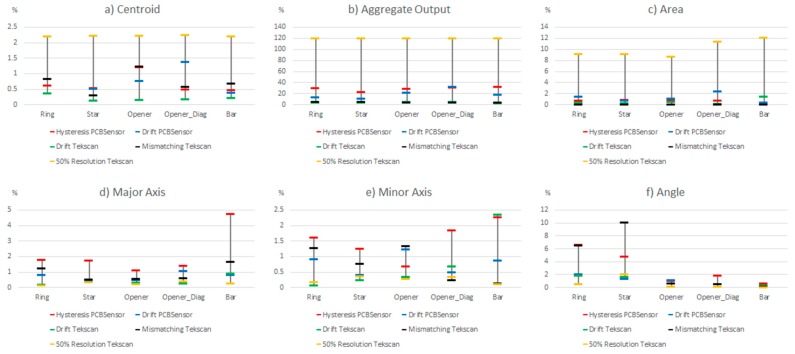
Comparison of the influence of different non idealities on the parameters of Table 4 (percentile 80%).

**Figure 15:**
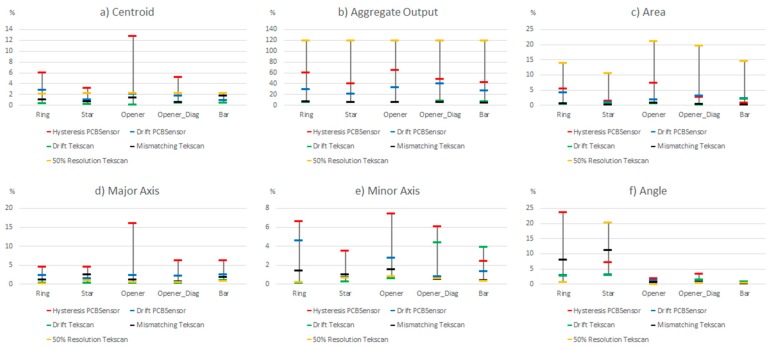
Comparison of the influence of different non idealities on the parameters of Table 4 (maximum deviation).

As mentioned in the introduction, the goal of this paper is to see the influence of common non-idealities in certain high level parameters. Besides some partial conclusions previously provided, this section aims to compare the effect of the different sources of error. For this purpose, some data is summarized and rearranged in Table 10 and Table 11. Moreover, the same information is shown graphically in Figure 14 and Figure 15. These tables and figures display the relative variations caused by hysteresis, drift, mismatching, and limited spatial resolution on the parameters in Table 4 computed for the objects in Figure 2a. Table 11 and Figure 15 are obtained from the full set of data, while Table 10 and Figure 14 show the results from the percentile 80 of the data. Note from previous sections that large variations were found for small forces. Tactile images show that the profile of the objects was not well defined yet for these small forces, so the conclusions cannot be reliable enough. Therefore the discussion focuses mainly of the results obtained for the percentile 80 of the data to eliminate this effect and the outliers. In the following, every parameter in Table 4 is discussed.

**Table 10:** Comparison of the influence of different non idealities on the parameters of Table 4 (percentile 80%).

CENTROID	Ring	Star	Opener	Opener Diag.	Bar
Hysteresis PCB Sensor	0.62	0.54	1.21	0.49	0.48
Drift PCB Sensor	0.82	0.51	0.78	1.38	0.38
Drift Tekscan	0.35	0.12	0.14	0.18	0.21
Mismatching Tekscan	0.83	0.30	1.23	0.58	0.68
50% Resolution Tekscan	2.21	2.22	2.22	2.25	2.21
25% Resolution Tekscan	6.65	6.49	6.58	6.69	6.59
AGGREGATE OUTPUT	Ring	Star	Opener	Opener Diag.	Bar
Hysteresis PCB Sensor	29.36	23.19	29.20	31.50	31.72
Drift PCB Sensor	13.43	11.30	21.24	32.34	18.24
Drift Tekscan	4.02	3.68	4.77	4.43	2.63
Mismatching Tekscan	5.14	5.13	3.85	3.99	3.67
AREA	Ring	Star	Opener	Opener Diag.	Bar
Hysteresis PCB Sensor	0.78	0.86	0.86	0.78	0.39
Drift PCB Sensor	1.41	0.70	1.09	2.42	0.39
Drift Tekscan	0.39	0.27	0.55	0.20	1.41
Mismatching Tekscan	0.00	0.00	0.00	0.00	0.00
50% Resolution Tekscan	9.10	9.08	8.69	11.45	12.11
25% Resolution Tekscan	28.91	28.52	25.88	34.79	31.17
MAJOR AXIS	Ring	Star	Opener	Opener Diag.	Bar
Hysteresis PCB Sensor	1.78	1.72	1.10	1.38	4.73
Drift PCB Sensor	0.80	0.40	0.49	1.06	0.81
Drift Tekscan	0.17	0.33	0.32	0.26	0.88
Mismatching Tekscan	1.22	0.51	0.58	0.61	1.67
50% Resolution Tekscan	0.12	0.33	0.24	0.39	0.27
25% Resolution Tekscan	0.64	1.44	0.48	0.99	0.43
MINOR AXIS	Ring	Star	Opener	Opener Diag.	Bar
Hysteresis PCB Sensor	1.62	1.26	0.68	1.85	2.28
Drift PCB Sensor	0.91	0.40	1.23	0.49	0.87
Drift Tekscan	0.07	0.24	0.33	0.67	2.35
Mismatching Tekscan	1.27	0.76	1.34	0.23	0.12
50% Resolution Tekscan	0.16	0.37	0.27	0.34	0.12
25% Resolution Tekscan	1.29	2.04	2.10	1.35	1.13
ANGLE	Ring	Star	Opener	Opener Diag.	Bar
Hysteresis PCB Sensor	6.61	4.77	1.02	1.88	0.65
Drift PCB Sensor	2.08	1.34	1.15	0.57	0.09
Drift Tekscan	1.80	1.63	0.16	0.11	0.36
Mismatching Tekscan	6.55	10.08	0.63	0.52	0.11
50% Resolution Tekscan	0.49	2.06	0.09	0.12	0.02
25% Resolution Tekscan	1.67	26.52	0.13	0.32	0.14

**Table 11:** Comparison of the influence of different non idealities on the parameters of Table 4 (maximum deviation).

CENTROID	Ring	Star	Opener	Opener Diag.	Bar
Hysteresis PCB Sensor	6.04	3.28	12.76	5.28	0.94
Drift PCB Sensor	2.82	1.09	2.16	1.81	0.91
Drift Tekscan	0.41	0.23	0.16	0.53	0.47
Mismatching Tekscan	1.12	0.72	1.42	0.64	1.81
50% Resolution Tekscan	2.21	2.23	2.24	2.25	2.21
25% Resolution Tekscan	6.66	6.73	6.59	6.72	6.95
AGGREGATE OUTPUT	Ring	Star	Opener	Opener Diag.	Bar
Hysteresis PCB Sensor	60.60	40.72	65.77	48.44	43.29
Drift PCB Sensor	29.56	21.58	33.41	40.30	27.88
Drift Tekscan	5.88	5.89	6.44	8.08	6.79
Mismatching Tekscan	7.00	6.11	6.60	5.60	5.10
50% Resolution Tekscan	120.00	120.00	120.00	120.00	120.00
25% Resolution Tekscan	176.47	176.47	176.47	176.47	176.47
AREA	Ring	Star	Opener	Opener Diag.	Bar
Hysteresis PCB Sensor	5.47	1.56	7.42	2.73	0.78
Drift PCB Sensor	4.30	1.17	1.95	3.13	2.34
Drift Tekscan	0.49	0.68	0.68	0.29	2.15
Mismatching Tekscan	0.59	0.20	0.78	0.39	0.29
50% Resolution Tekscan	13.96	10.55	21.09	19.63	14.65
25% Resolution Tekscan	33.11	29.98	44.73	41.02	39.65
MAJOR AXIS	Ring	Star	Opener	Opener Diag.	Bar
Hysteresis PCB Sensor	4.68	4.61	16.03	6.33	6.23
Drift PCB Sensor	2.34	1.47	2.37	2.19	2.52
Drift Tekscan	0.32	0.35	0.39	0.32	1.10
Mismatching Tekscan	1.28	2.51	1.18	0.67	1.93
50% Resolution Tekscan	0.20	1.09	0.48	0.53	0.91
25% Resolution Tekscan	0.73	2.15	1.11	1.29	1.46
MINOR AXIS	Ring	Star	Opener	Opener Diag.	Bar
Hysteresis PCB Sensor	6.62	3.53	7.46	6.08	2.45
Drift PCB Sensor	4.61	0.76	2.80	0.83	1.34
Drift Tekscan	0.12	0.31	0.63	4.39	3.91
Mismatching Tekscan	1.45	1.04	1.58	0.57	0.42
50% Resolution Tekscan	0.24	0.72	0.83	0.60	0.37
25% Resolution Tekscan	1.36	2.11	2.96	1.45	1.24
ANGLE	Ring	Star	Opener	Opener Diag.	Bar
Hysteresis PCB Sensor	23.62	7.25	1.94	3.52	0.79
Drift PCB Sensor	2.97	3.26	1.39	1.41	0.34
Drift Tekscan	2.81	3.08	0.27	1.46	0.85
Mismatching Tekscan	8.14	11.22	0.67	0.70	0.17
50% Resolution Tekscan	0.70	20.27	0.14	0.40	0.37
25% Resolution Tekscan	2.31	40.54	0.52	0.36	0.18

Centroid (contact location): The largest variation is due to the limited spatial resolution. Note that the 50% lowering of resolution results in a sensor whose spatial resolution is still higher than that of the sensor based on PCB. The change in the centroid location for this reason is quite large in comparison with the other sources of error. The latter affect the result to a similar degree, generally speaking, except the drift of the commercial sensor that has hardly any influence.

Aggregate output (contact force): The hysteresis and drift of the PCB based sensor have a large effect, so the estimation of this parameter has a significant error with this sensor. The variation caused by the mismatching and drift of the commercial sensor are much smaller, but quite significant when compared to the effect on other parameters. It makes no sense to observe the effect of the limited spatial resolution here, because it introduces only a scale factor in all cases.

Area (contact size): The largest variation is produced by the limited spatial resolution, and it is quite high. The drift of the PCB based sensor has a moderate influence, and that of the hysteresis is slightly smaller, while the drift of the commercial sensor has a small effect on this parameter. Moreover, mismatching has no effect at all if percentile 80 is considered because once the force is high enough, the object is well defined in the tactile image and the area does not change. If we consider the whole set of data it has a small influence.

Major and minor axes (contact shape and size): Here the largest variation is due to hysteresis, but the change due to mismatching is close, generally speaking. The error caused by the drift of the PCB based sensor is also quite similar but smaller, while the drift of the commercial sensor has a small impact. It is also worth highlighting that limited spatial resolution changes these results very little.

Angle (contact orientation): As mentioned in previous sections, the effect of the considered sources on the orientation is larger in objects with radial symmetry than that observed in objects with axial symmetry. The hysteresis has quite a high influence on this parameter but the effect of mismatching is even larger. The impact of drift is smaller but not negligible and it is quite close in both the commercial and the PCB based sensor. The resolution has a similar influence but the change is larger in the star than in the ring, which seems logical.

## 6. Conclusions

As stated above, the goal of this paper is to find out to what extent some significant parameters in manipulation with robotic hands, obtained from tactile sensors, are affected by the main limitations and errors of these sensors. An error-prone, low cost PCB sensor was used in the experiments, as well as a commercial one. In summary, the hysteresis and drift of the simple low-cost PCB based sensor cause error mainly in the estimation of the contact force and also of the contact shape (major and minor axes). However, the influence of the mismatching of the commercial sensor is similar in the estimation of the contact shape. On the other hand, the error in the orientation of objects with axial symmetry is small in all cases, while it can be large if the object has radial symmetry (though it can be less important in this case). In addition, the impact of the limited spatial resolution is far from being negligible when the contact location (centroid) and size (area) are estimated. We can conclude that there are sources of error which are difficult to reduce in the case of robotic hands such as the mismatching and others accepted such as limited spatial resolution, that have a similar impact on the high level parameters used in robotics to other limitations like hysteresis or drift. It seems that simple low cost sensors are good enough to provide information related to spatial distribution like orientation, shape or contact location, though are not good at providing other information like contact force, which is just the aggregation of the output of all taxels.
